# Young Floating Population in City: How Outsiderness Influences Self-Esteem of Rural-to-Urban Migrant Children in China?

**DOI:** 10.3390/ijerph19031863

**Published:** 2022-02-07

**Authors:** Bo Zhou, Yumeng Zhong

**Affiliations:** 1 Public Administration School, Guangzhou University, Guangzhou 510006, China; 2 School of Sociology and Anthropology, Sun Yat-sen University, Guangzhou 510275, China

**Keywords:** rural-to-urban migrant children, self-esteem, outsiderness, shared home, migrant sponsored school, discrimination

## Abstract

While scholars note that rural-to-urban migrant children in China tend to have worse mental health than urban-born children, insufficient attention has been paid to understanding this mechanism beyond the Hukou system and the urban-rural dual structure. Using data from China’s Nine-City Survey of Migrant Children, this study reveals that perceptions of being a temporary visitor and an outsider in the city have strong negative effects on migrant children’s self-esteem. Regression analysis shows that migrant children sharing a kitchen with other families, studying in migrant-sponsored schools instead of regular schools for local children and perceiving discrimination from local peers tend to have lower self-esteem.

## 1. Introduction

According to China’s census data, the size of the floating population, or temporary migrants working or residing in a city or county different from their household registration (Hukou), has increased from 221 million in 2010 to 376 million in 2020 [1] of which mostly are rural-to-urban migrant workers and their family members. The number of migrant children in China increased dramatically from 19 million in 2000 to 35 million in 2010 [2]. Probably due to the negative influence of poor conditions in receiving communities in cities, the number of migrant children dropped slightly to 34.3 million between 2010 and 2015 despite the rapid growth of parental migration during this period [3].

Studies involving rural-to-urban migrant children in China found that these young people floating in cities are more likely to become child laborers [4], face many problems in adapting to city life [5,6], and are more vulnerable to physical and mental health issues [7,8]. One of these important issues that deserves further study is the low self-esteem problem of migrant children. On the one hand, low self-esteem is are believed to be highly correlated with other psychological problems, such as distress and depression [9]. On the other hand, low self-esteem can lead to worse childhood development and has long-term consequences [10].

The fundamental cause of the low self-esteem of rural-to-urban migrant children might be the structural difference between rural and urban China. For over 60 years, there has been a wide income gap between rural and urban residents in China. The Household Registration System (Hukou) has constrained rural-to-urban migration and thus sustained the urban-rural dual structure. Rural residents have long been bonded to low wage agricultural jobs because of their rural Hukou. There are extremely limited educational resources and job opportunities in the rural areas, which further expands the income gap between rural and urban residents.

While most studies focus on the impact of rural-urban economic inequality embedded in the Hukou system, insufficient research has been done on how such structural inequality affects the mental well-being of migrant children through emotional impact. Due to budget limitations, rural-to-urban migrant families usually share an apartment with other families, so migrant children residing in cities rarely have their own rooms to study or entertain at home [11]. Migrant children face extra obstacles in the public school enrollment process and sometimes need to turn to migrant sponsored schools [12]. We believe such experiences would keep reminding migrant children about the fact that they are just temporary visitors and outsiders in the city, reducing their belongingness to their current home, community and school. Hence we aim to examine how rural-urban inequality reinforces migrant children’s sense of outsiderness, that is, the sense of themselves being outside of the world of others, and consequently leads to low self-esteem issues. Through moving beyond traditional scope on effects of material difference, our study might be able to offer a deeper understanding of the linkage between objective conditions and subjective well-being of migrant children. Based on the China’s Nine-City Migrant Children collected nation-wide, findings of this study would also possess higher generalizability than prior regional or single-city studies on Chinese migrant children [5,10,11,13].

In the next section, we review prior studies on influential factors of children’s self-esteem. After that, we examine the effects of living conditions, neighborhood, type of school and discrimination on migrant children’s self-esteem using the Nine-City Data. Finally, we conclude about how experiences in urban homes, urban neighborhoods and urban schools lead to low self-esteem problem of rural-to-urban migrant children and present several corresponding suggestions to policy makers.

## 2. Background

Self-esteem usually refers to the enduring feelings of people about themselves (global/trait self-esteem), the evaluation people made on their own abilities and attributes (self-evaluation), or the momentary feeling of self-worth that arises from a positive or negative outcome (state self-esteem) [14]. There are four main sources of self-esteem: social comparisons, reflected appraisals, self-attributions, and psychological centrality [15]. Social comparison theory suggests that humans are driven by instinct to evaluate themselves, usually through comparing their opinions and abilities with those of others. As migrant children grow older, they are more likely to compare their clothes and living conditions with peers in school and become more aware of the differences related to family economic status [16].

In the case of China, economic and cultural differences between people of rural and urban origins have remained for decades under the urban-rural dual structure. Noticing the difference between themselves and local children, migrant children would be more conscious of the fact that they are only outsiders temporarily staying in the city and may even feel that they are inferior to local children. Even though one’s sense as an outsider in the world is an inevitable thing in one’s life, outsiderness can have mental health consequences if not dealt with properly [17], especially for these migrant children in an unfamiliar place.

### 2.1. Shabby Home in City

Prior works show that a family’s economic conditions have great influence on children’s self-esteem [15,18]. Being one of the important indicators of family wealth, living conditions have a clear influence on rural-to-urban migrant children’s mental health and self-esteem. Youths generate their self-esteem through comparing themselves to those around them, based on their social similarity/dissimilarity with their reference groups [19].

Studies in China often find that rural-to-urban migrants tend to have worse living conditions than urban locals and even rural non-migrants. Since most rural-to-urban migrants tend to have lower social economic status than urban residents and cannot afford the high rent in a city, they often begin their city lives in shabby apartments located in disadvantaged neighborhoods known as the “urban villages” [7]. Urban villages are the remaining parts of suburban villages that were merged by expanding cities. Compared to apartments in surrounding urban areas, apartments in the urban villages have much lower rents, which attract the rural-to-urban migrants. A typical apartment occupied by rural-to-urban migrants has far more sanitation problems than those possessed by city locals [20]. During a study on migrant families with children, Liu shows that 60% of the migrant families lived in rooms smaller than 108 square feet, which is much smaller than the common dwellings in rural China [11].

The poor living conditions in cities have led to uncomfortable lives for children who migrated with their migrant worker parents. The typical overcrowded apartment for rural-to-urban migrant families often does not include a children’s room or studying room, and children have to study and play in the living room (that is, if there is one) while parents and other renters are walking around [11]. In an ethnographic study on people from a village in Jinjiang to Hong Kong city, Lin notes that migrant children tend to avoid inviting any local peers to their home, since they rarely view their shabby place in the city as their home [21]. Wang points out that migrant children tend to lack belongingness of their home because it’s not functioning properly as the home for these children [22].

In this study, we test two hypotheses on how living conditions in the city influence migrant children’s self-esteem. The first one is about the functional inadequacies of their home in the city:

**Hypothesis** **1a** **(H1a).** *Migrant children without an independent children’s room tend to have lower self-esteem*.

The second is about effect of the signs reminding migrant children that they are not living in their “home” but are just outsiders temporarily staying in the city:

**Hypothesis** **1b** **(H1b).** *Migrant children sharing a housing unit with non-relative renters tend to have lower self-esteem*.

### 2.2. Urban Life in Urban Villages

Jencks and Mayer review works on the consequences of growing up in poor neighborhoods and conclude that the neighborhood has a significant influence on children’s education attainment, cognitive skills, likelihood of committing a crime, likelihood of becoming a single mother, and labor market success [23,24]. Most scholars believe that “good” neighbors lead to better performance in children, because peers in poor neighborhoods are more likely to spread problematic behavior (epidemic model) [25], neighborhood role models and monitoring help children to socialize (collective socialization model) [26], and neighborhood institutions influence children (institutional model) [27]. However, some scholars predict that “good” neighbors do not necessarily lead to good performance among children. They think children can be discouraged when they compare themselves with good neighbors (relative deprivation model) [28], and “good” neighbors with higher social-economic status are also more likely to win the competition for neighborhood resources (competition model) [29].

Many scholars strongly believe that living in a disadvantaged neighborhood can lead to mental problems and low self-esteem for children [30,31,32]. Raviv’s research done in Israel demonstrates that exposure to violence in a neighborhood, either as a victim or as a witness, would lead to significantly higher internalizing, externalizing, and total behavior problem scores [31]. Plybon and Kliewer reveal that compared to black children living in low crime rate districts, black children living in moderate crime rate districts clearly have more external behavior problems [32]. Other studies failed to find clear connections between neighborhood crime and children’s mental health [33]. A panel study done in black and Hispanic neighborhoods showed that perceived crime has clear influence on the mental well-being of adults, but not on the mental health of children.

Scholars argue that the urban villages where many rural-to-urban migrant children reside share many characteristics with urban slums, such as a high crime rate and scarce neighborhood resources [34]. Even though the disadvantaged neighborhoods can have a negative influence on migrant children’s well-being, few scholars in China have studied this issue. Only a few studies were conducted on the linkage between neighborhood public order and migrant children’s outdoor activities [35]. Given the fact that many rural-to-urban migrant children reside in neighborhoods with poor environment and high risks of being involved in crimes, this study examines the following two hypotheses on the effect of neighborhood conditions:

**Hypothesis** **2a** **(H2a).** *Migrant children who dislike their current neighborhoods tend to have lower self-esteem*.

**Hypothesis** **2b** **(H2b).** *The more frequent migrant children witness other migrant children being arrested in their neighborhoods, the lower self-esteem they tend to have*.

### 2.3. Studying in “Special” Schools for Migrants

The Hukou system in China sets a concrete barrier on school enrollment for migrant children [12]. The regular schools that urban local children study in include public schools and private schools. Public schools offer better education with low tuition fees, but have strict requirements on students’ Hukou and only a small portion of migrant children were able to be enrolled in public schools. Meanwhile, private schools do not require a local Hukou but in general require rather high tuition fees. An alternative option for migrant children are migrant sponsored schools, which are operated by rural-to-urban migrants and have no requirement on Hukou. Typical migrant sponsored schools charge extremely low tuition fees, but often lack reliable teachers and necessary educational resources [12]. In fact, many migrant sponsored schools have been padlocked by city governments in China for failing to meet minimum requirements of schools. As a large proportion migrant children can’t afford the tuition fees of private schools and lack the social capital to be enrolled in public schools, we find it necessary to examine how fondness of school and school type affect migrant children’s self-esteem:

**Hypothesis** **3a** **(H3a).** *Migrant children who dislike their current schools tend to have lower self-esteem*.

**Hypothesis** **3b** **(H3b).** *Migrant children in migrant sponsored schools tend to have lower self-esteem than those in public or private schools*.

### 2.4. Being Looked Down Upon by Local Peers

Awareness of discrimination is a key component of group identity [36]. After experiencing discrimination, migrant children may realize that they belong to a minority group being dominated by the majority. These minority group memberships, along with frequent marginalization, can affect the way they view and appraise themselves [37]. The effects of discrimination on migrant children’s self-esteem are complex. Paradies reviewed twenty-six studies on the relation between racial discrimination and self-esteem, nine of them found a negative association, four of them found a positive association, and thirteen of these studies found no significant association between discrimination and self-esteem [38]. Studies conducted in North America and Europe show that discrimination can lead to distress and low self-esteem among immigrant children and young descendants of immigrants [39,40]. Teenagers socialized to be aware of and respond proactively to racism have been found to have higher self-esteem [41,42].

Most of the rural-to-urban migrant children in China are part of the ethnic majority (Han nationality), and the discrimination they face is mainly caused by the income difference embedded in the Hukou system. Among city locals, rural migrants are often stereotyped as poor, dull and rustic people who brought crime into the cities, so discrimination and exclusion are clearly affecting rural-to-urban migrants’ mental well-being [12,43]. Hence, we examine the following two hypotheses on the effect of discrimination on rural-to-urban migrant children:

**Hypothesis** **4a** **(H4a).** *Migrant children being more afraid of peer discrimination from local children tend to have lower self-esteem*.

**Hypothesis** **4b** **(H4b).** *Migrant children complained about being discriminated to guardians tend to have lower self-esteem*.

## 3. Data and Methods

This study is mainly based on the China’s Nine-City Survey of Migrant Children Survey data collected by the United Nations Children’s Fund, China’s National Working Committee on Children and Women, and the China’s Children Center in 2002. This survey was designed to examine the wellness of migrant children in nine Chinese cities, of which three cities of different sizes are located in the Eastern coastal region and the other six cities are located in inland China (for details, see Appendix A). Thus Nine-City Survey data allow scholars to generate a general picture of the well-being of migrant children in China. The survey only selected respondents who hold rural Hukous, stayed in cities for at least half a year, and have children under age 18. A systematic random sampling of migrant households was conducted, controlling for the number in the sample within each parental occupation group and age group. This study was conducted following research ethical codes and all respondents participated in the survey on a voluntary basis.

The Nine-City Survey provides information of rural-to-urban migrant children on various subjects and hence allows for analysis of the influences of living conditions, neighborhood conditions, school conditions and discrimination on the self-esteem of migrant children. Surveys on the self-esteem of migrant children are often constrained to one city or one province, while the data in the Nine-City Survey allows us to control for regional differences. The Nine-city data set with a sample size of 1734 also allows for more types of in-depth analysis.

The dependent variable for this study is the self-esteem score of migrant children, which is measured with the Rosenberg Self-Esteem Scale (RSES). By 2005 the RSES has already been translated into 28 languages and applied in 53 nations [44]. While the RSES factor structure was largely invariant across nations, the meaning of the ten statements could be altered in certain versions of translation. The statement “I wish I could have more respect” (item 9 in Nine-City Survey) represents a positive attitude in the Chinese version of RSES, which sounds more like “I will try to win more respect for myself” in Chinese [45,46]. Following the practice of other Chinese scholars, we treat item 9 as a positive item in this study (Appendix B). A continuous index on self-esteem is generated by adding up the scores of the 10 Rosenberg self-esteem items (reverse scoring applied to the 4 negative items).

The independent variables include migrant children’s gender (0 = female, 1 = male), age, family structure (a 4-category nominal variable: 1 = Living with both parents, 2 = Living with mother only, 3 = Living with father only, 4 = Not living with parents), highest educational attainment of guardians (a 3-category ordinal variable: 1 = Below high school, 2 = High school or junior college, 3 = college and beyond), region (0 = Western or central city, 1 = Eastern city) guardians’ time spent with children each day (unit: min), living conditions, neighborhood conditions, school conditions, and discrimination.

Family structure is a categorical variable indicating whether a migrant child is residing in a city with both parents, with one parent, or with other relatives. Two indicators of living conditions are being selected: one is the kitchen condition, which also indicates whether a respondent is sharing a home with other families (1 = Private kitchen, 2 = Shared kitchen, 3 = No kitchen), and the other one is whether there is a separate room for the children. The two variables indicating neighborhood conditions are how much they like current neighborhoods and how often they witness migrant children being arrested in their neighborhood. School conditions are measured by two dichotomous variables: one is whether a respondent is enrolled in a migrant-sponsored school, and the other one is whether a respondent likes the current school. There are two variables measuring discrimination faced by migrant children: the first is how much respondents worry about discrimination from local children, and the other one is whether they reported being discriminated to guardians.

A set of six OLS linear regression models are used in this study to analyze influential factors of the self-esteem score of rural-to-urban migrant children in China (for univariate analysis, see Appendix C). The first model is a base model including only indicators of basic demographic characters and family variables. In additional to variables included in the base model, the second to fifth models each include a set of indicators on living conditions, neighborhood conditions, school conditions, and discrimination corresponding to Hypotheses 1 to 4. The last model is a full model that includes all independent variables used in the prior models.

## 4. Findings

The average age of migrant children included in the Nine-City Survey data is 13.91 (Table 1). The average self-esteem score of migrant children in these nine Chinese cities is 39.13. The living conditions of migrant children are rather undesirable, that is, about 18% of migrant families have to share a kitchen with other families, and 36.2% of families don’t have a kitchen at all. Only 37.8% of the migrant children have an independent room. About 11.2% of the migrant children occasionally witnessed migrant children being arrested in their neighborhoods, and 5.5% frequently witnessed migrant children being arrested. About 12.9% of migrant children were enrolled in the migrant-sponsored school. Most of the migrant children (73.6%) like their current schools, probably because the school conditions in cities are much better than that back in their home villages. While 38.7% of the migrant children worried about being looked down upon by local children, only 25.6% of the migrant children reported being discriminated against to their guardians.

Results of model 1 show that compared to migrant children living with both parents, those living with their father and not with their mother would have a 1.38 points lower self-esteem score (Table 2). Migrant children in a mother-only household or living with other relatives do not tend to have significantly lower self-esteem than those living with both parents. Migrant children in eastern cities that are in general more developed tend to have higher self-esteem scores than those in central and western China.

In Model 2, migrant children in families sharing kitchens with other families would have a one point lower self-esteem score than those in families possessing their own kitchens. Meanwhile, the difference between the self-esteem scores of migrant children without a kitchen and those with a private kitchen is not statistically significant. This indicates that migrant children tend to have lower self-esteem when they have to share their home with non-relative renters. Hence, Hypothesis 1a is supported by the regression result. Possession of an independent children’s room has positive effects on migrant children’s self-esteem, which is marginally significant, providing some support to Hypothesis 1b.

Results of Model 3 only partly support Hypothesis 2a and Hypothesis 2b. While migrant children who occasionally witness other migrant children being arrested in their neighborhoods and don’t like their neighborhoods a lot tend to have lower self-esteem, those who frequently witness other migrant children being arrested or dislike their neighborhoods a lot don’t have significantly lower self-esteem scores than those residing in neighborhoods with the best conditions. This finding indicates that the relationship between neighborhood conditions and migrant children’s self-esteem is non-linear. One possible explanation is that some migrant children living in neighborhoods with extremely poor conditions would view themselves as stronger and more adaptive persons than their peers and would thus feel proud of themselves.

In Model 4, migrant children who like their current schools tend to have a 1.03 point higher self-esteem score than those who don’t. Migrant children studying in migrant sponsored schools tend to have a 1.28 points lower self-esteem than those in public schools or private schools. Hence Hypothesis 3a that better school environment benefits migrant children’s self-esteem and Hypothesis 3b that being enrolled in “special” schools for migrant children lowers self-esteem are both supported by regression results.

Results of Model 5 indicate that concerns about discrimination have significant negative effects on self-esteem and provide supports to Hypothesis 4a. However, migrant children who complaint about being discriminated against to guardians don’t seem to have significantly lower self-esteem. A possible explanation for this phenomenon is that not all children experiencing discrimination choose to talk about it with guardians, but only those with a closer relationship with their guardians would do so. Moreover, discussing unpleasant experiences helps reduce its negative impact on mental well-being to some extent.

In Model 6, where all dependent variables have been included, the negative effects of sharing kitchens with other families, occasionally witnessing other migrant children in the neighborhood being arrested, studying in migrant-sponsored schools and worrying about discrimination from local peers remains to statistically significant at the 0.05 level. This indicates that signs of being outsiders in the city indeed impair the self-esteem of rural-to-urban migrant children.

## 5. Discussion

While this current study presents the negative effect of outsiderness on the self-esteem of migrant children, it is not without limitation. First, the Nine-City Survey doesn’t include a direct measurement of outsiderness. We managed to bypass data limitations and expand our vision through using less direct measures related to housing, neighborhood and school conditions. Nevertheless, we do recognize the importance of direct measurements and expect to include both direct and indirect measurements of outsiderness in a future study to present an even more comprehensive view of the effect of outsiderness. Second, without qualitative data collected through in-depth interviews or focus groups, we are not absolutely sure that the indirect measurements have actually captured the signs of outsiderness. When regression results show that migrant children residing in an apartment sharing a kitchen with other families tend to have lower self-esteem, we treat sharing kitchen as a sign of outsiderness that can impair children’s sense of ownership of their apartment in the city; however, perhaps children were worrying about something else, such as food insecurity. We expect that in the future there will be studies that combine qualitative and quantitative methods and offer a deeper understanding of the subjective aspect of migrant children.

## 6. Conclusions

Despite the large body of work on migrant children’s well-being, few studies have been done on how migration leads to mental health issues through emotional trajectories. Our current study examining the experiences of rural-to-urban migrant children in China provides a piece of information on this critical issue. In line with many prior studies on this issue, our study finds that migrant children tend to have lower self-esteem because of their dwellings, neighborhoods and schools are in worse conditions than those of local children. But beyond this, our study also demonstrates that sharing an apartment with non-relative renters, occasionally witnessing other migrant children being arrested, being enrolled in schools sponsored by migrant workers and sensing discrimination from local peers would lead to lower self-esteem for migrant children. These results indicate that migrant children with a stronger sense of outsiderness and weaker sense of belonging in cities tend to have lower self-esteem. Our findings on the negative psychological effect of outsiderness would help strengthen the theoretical linkage between objective conditions and subjective well-being for migrant children.

Our study reveals that unequal opportunity and stratified identity embedded in the Hukou system have severely affected the self-esteem of rural-to-urban migrant children. This implies that an effective way of increasing rural-to-urban migrant children’s mental well-being is to offer their families the equivalent access to social welfare as the urban locals. Allowing migrant families to apply for low-rent apartments can be a good start, since this would lead to significant improvement of migrant children’s dwelling and neighborhood conditions. Even though many cities in China have built state-owned low-rent apartments, most of which are only accessible for city residents with local Hukou [47]. For instance, Shijiazhuang city only accepts applications from residents who have owned local Hukou for more than three years. Furthermore, rural-to-urban migrant children should be allowed to equally benefit from educational resources in the city. In many cities, children with rural Hukou can’t be enrolled in public schools even if they reside in good school districts [48,49]. Most importantly, granting rural migrant families more equal access to housing and educational welfare can weaken the institutional division and significantly relieve migrant children’s sense of being outsiders in the city, hence effectively improving their mental well-being.

## Figures and Tables

**Table 1:** Descriptive Statistics of Rural-to-Urban Migrant Children.

Continuous	Mean	SD
Self-Esteem Score	39.13	5.43
Age	13.91	2.09
Minutes Spent Attending Child Every Day	58.08	41.12
Categorical		Percentage
Gender	Female	44.3%
	Male	55.7%
Family Structure	Living with both parents	87.1%
	Living with mother only	5.4%
	Living with father only	4.4%
	Not living with parents	3.1%
Education	Below high school	74.1%
	High school or junior college	24.2%
	college and beyond	1.7%
Region	Eastern	29.9%
	Western or central	70.1%
Kitchen	Private kitchen	45.6%
	Shared kitchen	18.2%
	No kitchen	36.2%
Children’s Room	Yes	37.8%
	No	62.2%
Fondness of Current Neighborhood	Like a lot	18.2%
	Like	42.0%
	Neutral	24.4%
	Dislike	10.6%
	Dislike a lot	4.8%
Migrant Children Arrested	Never	83.3%
	Occasionally	11.2%
	Frequently	5.5%
School Types	Regular	87.1%
	Migrant school	12.9%
Like Current School	Yes	73.6%
	No	26.4%
Worry about Discrimination	Not at all	58.8%
	A little	38.8%
	A lot	2.4%
Complain about discrimination to guardians	Yes	74.4%
	No	25.6%

**Table 2:** OLS Regression Models Predicting Migrant Children’s Self-Esteem Score.

	Model 1	Model 2	Model 3	Model 4	Model 5	Model 6
Age	−0.03	−0.02	−0.01	0.10	−0.03	0.08
Male	−0.34	−0.35	−0.28	−0.43	−0.40	−0.42
Family structure (those living with both parents as the reference group)						
Living with mother only	−0.98	−0.93	−0.78	−0.04	−0.63	0.08
Living with father only	−1.38 *	−1.31 *	−1.11 ^+^	−1.47 ^+^	−0.89	−0.93
Not living with parents	1.11	1.15	1.05	2.25	1.29	3.11 ^+^
Guardian’s time spent with child every day (unit: min)	0.00	0.00	0.00	0.00	0.00	0.00
Highest level of education of guardian (below high school as the reference group)						
High school or junior college	−0.24	−0.17	−0.21	0.00	−0.10	0.33
college and beyond	−1.17	−0.97	−1.24	−0.78	−0.84	−0.49
Eastern region	0.86 **	0.84 **	0.72 *	1.05 **	0.60 *	0.58 ^+^
Kitchen condition (those with a private kitchen as the reference group)						
Shared a kitchen	-	−1.04 **	-	-	-	−1.47 ***
No kitchen	-	0.02	-	-	-	0.02
Independent children’s room	-	0.51 ^+^	-	-	-	0.24
Witnessing migrant children being arrested (those never witnessed as the reference group)						
Occasionally	-	-	−1.69 ***	-	-	−0.96 *
Often	-	-	−0.69	-	-	−0.26
Preference of neighborhood (those like current neighborhoods a lot as the reference group)						
Like	-	-	−1.08 **	-	-	−1.22 **
Neutral	-	-	−1.12 **	-	-	−0.60
Dislike	-	-	−1.31 **	-	-	−1.45 *
Dislike a lot	-	-	−0.15	-	-	0.31
Like current school	-	-	-	1.03 **	-	0.73 *
In migrant school	-	-	-	−1.28 **	-	−0.85 ^+^
Worry about discrimination (those do not worry at all as the reference group)						
A little	-	-	-	-	−2.17 ***	−1.62 ***
A lot	-	-	-	-	−2.58 ***	−1.73 **
Complain about discrimination to guardian	-	-	-	-	0.42	0.28
Constant	39.61 ***	39.49 ***	41.16 ***	40.56 ***	37.45 ***	39.85 ***
N	1734	1734	1659	1734	1341	1277
R^2^	0.011	0.018	0.046	0.025	0.022	0.060

Note: 2-sided *t*-test, ^+^ sig 0.1, * sig 0.05, ** sig 0.01, *** sig 0.001.

## Data Availability

The data presented in this study are available on request from the author.

## Appendix A

**Table A1:** Population and Sample Size of Each Sampling Point.

	Large	Medium	Small
**Coastal**	Beijing	Shenzhen	Shaoxing
Pop.	13.82 million	7.01 million	4.30 million
Samp.	1200	843	300
**Central**	Wuhan	Jilin	Zhuzhou
Pop.	8.31 million	4.49 million	3.58 million
Samp.	1200	500	300
**Inland**	Chengdu	Xianyang	Yining
Pop.	11.24 million	4.83 million	0.35 million
Samp.	1200	500	300

Note: The sampling unit is household.

## Appendix B

**Table A2:** Ten-Item Rosenberg Self-Esteem Scale from Nine-City Survey Data.

	Strongly Disagree	Disagree	Neutral	Agree	Strongly Agree
1. I feel that I am a person of worth, at least on an equal plane with others.	71	166	374	612	511
	4.1%	9.6%	21.6%	35.3%	29.5%
* 2. I certainly feel useless at times.	805	603	192	44	44
	46.4%	34.8%	11.1%	5.2%	2.5%
3. I feel that I have a number of good qualities.	25	111	431	802	365
	1.4%	6.4%	24.9%	46.3%	21.0%
* 4. All in all, I am inclined to feel that I am a failure.	888	510	165	103	68
	51.2%	29.4%	9.5%	5.9%	3.9%
5. I am able to do things as well as most other.	55	62	207	686	724
	3.2%	3.6%	11.9%	39.6%	41.8%
* 6. I feel I do not have much to be proud of.	245	501	471	391	126
	14.1%	28.9%	27.2%	22.5%	7.3%
7. I take a positive attitude toward myself.	32	154	452	605	491
	1.8%	8.9%	26.1%	34.9%	28.3%
8. On the whole, I am satisfied with myself.	38	154	315	679	548
	2.2%	8.9%	18.2%	39.2%	31.6%
9. I wish I could have more respect for myself.	81	80	150	501	922
	4.7%	4.6%	8.7%	28.9%	53.2%
* 10.At times I think I am no good at all.	726	581	198	147	82
	41.9%	33.5%	11.4%	8.5%	4.7%

N = 1734, Cronbach’s Alpha = 0.594; Note: questions with * are the negative attitude questions; Chinese version of item 9 is a positive notion, sounds more like “I will earn more respect for myself”.

## Appendix C

**Table A3:** Univariate OLS Analyses for Migrant Children’s Self-Esteem Score.

	Coef.	95% CI		*p*
Age (year)	−0.017	−0.140	0.105	0.781
Male	−0.350	−0.865	0.165	0.183
Family structure (vs. living with both parents)				0.0031
Living with mother only	−1.321	−2.451	−0.192	0.022
Living with father only	−1.502	−2.751	−0.253	0.018
Not living with parents	4.126	1.914	6.338	0
Time with child (minute)	0.005	−0.002	0.011	0.165
Guardian’s education (vs. below high school)				0.365
High school or junior college	0.585	−0.274	1.444	0.182
college and beyond	0.736	−2.029	3.501	0.602
Eastern region (vs. western and central)	0.986	0.429	1.543	0.001
Kitchen (vs. family with a private kitchen)				0.0005
Shared a kitchen	−1.410	−2.117	−0.703	0
No kitchen	−0.352	−0.919	0.216	0.224
With children room (vs. without)	0.544	0.017	1.072	0.043
Migrant children being arrested (vs. never)				0
Occasionally	−1.970	−2.780	−1.161	0
Often	−0.898	−2.014	0.218	0.115
Preference of neighborhood (vs. like a lot)				0.0023
Like	−1.179	−1.895	−0.463	0.001
Neutral	−1.460	−2.250	−0.670	0
Dislike	−1.544	−2.530	−0.559	0.002
Dislike a lot	−0.644	−1.955	0.666	0.335
Like current school (vs. dislike)	1.260	0.616	1.904	0
In migrant school (vs. in public or private)	−1.234	−2.083	−0.385	0.004
Worry about discrimination (vs. not at all)				0
A little	−2.007	−2.540	−1.474	0
A lot	−2.428	−3.410	−1.447	0
Complain about discrimination (vs. didn’t)	−0.418	−1.018	0.182	0.172
